# Malaria Parasites: The Great Escape

**DOI:** 10.3389/fimmu.2016.00463

**Published:** 2016-11-07

**Authors:** Laurent Rénia, Yun Shan Goh

**Affiliations:** ^1^Singapore Immunology Network, Agency for Science, Technology and Research (A*STAR), Singapore

**Keywords:** malaria, vaccine, escape mechanism, antibodies, T cells, immunosuppression, antigenic polymorphism, antigenic variation

## Abstract

Parasites of the genus *Plasmodium* have a complex life cycle. They alternate between their final mosquito host and their intermediate hosts. The parasite can be either extra- or intracellular, depending on the stage of development. By modifying their shape, motility, and metabolic requirements, the parasite adapts to the different environments in their different hosts. The parasite has evolved to escape the multiple immune mechanisms in the host that try to block parasite development at the different stages of their development. In this article, we describe the mechanisms reported thus far that allow the *Plasmodium* parasite to evade innate and adaptive immune responses.

## Introduction

Malaria, a disease caused by *Plasmodium* parasites and transmitted by *Anopheles* mosquitoes, remains one of the most deadly diseases. There are six species able to infect humans, namely, *Plasmodium falciparum, Plasmodium vivax, Plasmodium ovale, Plasmodium malariae*, and the zoonotic monkey malaria species *Plasmodium knowlesi* (1) and *Plasmodium cynomolgi* (2). Around 300 million cases of clinical malaria are recorded every year. Approximately, half a million deaths occur in Africa and are mainly due to *P. falciparum* infections (3).

*Plasmodium* parasites have a complex life cycle. It starts when sporozoites are inoculated into the dermis of the mammalian host by infected mosquitoes. Sporozoites are highly motile and a majority of them migrate from the skin to the capillaries for dissemination by the bloodstream (4, 5). They are retained in the liver where they transmigrate through Kupffer cells and hepatocytes before seeding in final hepatocytes (6, 7). Depending on the species of their mammalian hosts, sporozoites mature in 2–14 days. At maturity, budding vesicles called merosomes are released and are ruptured in the lung circulation where the merozoites are released, ready to infect red blood cells (RBC) (8). During the erythrocytic cycle, a fraction of parasites differentiates into male and female gametocytes which can be taken up during the feeding of an *Anopheles* mosquito. In the mosquito midgut, ookinetes, generated by the gametocyte fusion, cross the mosquito midgut wall and develop into oocysts. Sporozoites released from mature oocyst then migrate to the salivary gland, ready for the next round of infection during the mosquito’s next blood meal.

Malaria is a disease characterized by fever, headache, chills, sweating, and vomiting (9). Unlike viral or bacterial infections, the main indication of clinical malaria is the recurrent fever which varies between species. This is due to the release of parasite toxins into the bloodstream during the erythrocytic cycles of merozoite egress and reinvasion of erythrocytes. As the infection progresses, the number of RBC decreases and this may lead to severe anemia (10). In addition, RBC containing parasites such as *P. falciparum* can also sequester in deep tissues by cytoadhering to endothelial cells (11). This may cause organ failure, and is partly responsible for cerebral malaria. All these pathologies can eventually lead to death (9, 12, 13).

In the last two decades, the mortality to malaria has decreased substantially (3). This results from the combined efficacy of prevention measures, such as the use of insecticide-impregnated bednets, the development and use of rapid and easy to use diagnostic tools, and the potent artemisinin-based combinations therapies against the malaria parasites (14). However, this gain of lives might be temporary. In the recent years, all these interventions have shown some limitations. With the advent of decreased efficacy of artemisinin (15, 16), it is now clear that new drugs and other interventions should be developed (9, 17, 18). New drug families, such as spiroindolones (19, 20) and imidalopiperazines (21, 22) compounds, have shown promising results in phase II clinical trials in the recent years and have a great future ahead. However, a vaccine would be the most important tool in the armamentarium against malaria.

While vaccines have been readily developed for many bacterial and viral infections, there are currently no vaccines to protect against human parasites. The need to develop a vaccine to protect against malaria has been highlighted as early as the identification of the parasite in 1897 (23). There have been two schools of thought for the development an antimalarial vaccine. The first is based on the fact that naturally acquired immunity is often observed under field conditions. However, this immunity requires long period of time to develop. It first targets the disease and then the parasite (24). This immunity has been called premonition or relative immunity. It has been defined by Edmond Sergent in 1935 as “*a special type of immunity connected with the persistence of living germs in the organs of the immunized host*” (25). In other words, immunity is maintained as long as the host immunity is stimulated by the continuous or repeated parasite exposure. Understanding the mechanisms responsible for this premonition will help to develop a vaccine.

The other approach is based on the Jenner principle of vaccination, which was further exemplified by Louis Pasteur. Instead of letting Nature takes its course, this approach uses an inoffensive target as a formulation to induce an immune response in healthy individuals to protect against a subsequent infection. It might not be surprising, that the first attempt was reported by the same Sergent mentioned above, who was working in the Pasteur Institute in Algiers in Algeria. Sergent was able to partially protect birds from *Plasmodium lophurae*, an avian malaria parasite, using inactivated sporozoites (26). Decades later, in 1946, the first attempt in human was done by Heidelberger et al. using formalin-inactivated *P. vivax*-infected blood to immunize volunteers, however no protection was induced (27, 28). Jules Freund took it one step further by inventing the Freund adjuvant and combining the adjuvant with formalin-inactivated-blood infected with *P. lophurae* or *P. knowlesi*, a monkey malaria parasite. Freund was more successful in protecting ducks or rhesus monkeys, respectively (29, 30). However, because of its toxic side effects, the adjuvant could not be used in humans. Since then, various approaches and technologies have been used for vaccine development against the malaria parasites. Purified parasite preparation has been used as immunogens. Peptides, recombinant proteins, DNA plasmids, bacterial and viral vectors, and genetically modified malaria parasites, in combination with new adjuvants, have also been used as vaccine delivery systems (31). However, despite having more than thousands of pre-clinical trials in rodent and monkey models and more than 200 trials in humans, very few vaccine candidates have shown vaccine efficacy in human. The subunit vaccine, RTS,S, a chimeric molecule based on a large part of the circumsporozoite (CS) protein, the major surface protein of the sporozoite, fused to the S antigen of the hepatitis B virus, together with a strong adjuvant in the formulation, is the only candidate that has moved to Phase III clinical trials. However its efficacy was at best ~50% against infection or clinical disease (32, 33). So far, only whole-parasite based approaches have repeatedly shown high efficacy (34–36).

## Mechanisms of Immunity Against the Malaria Parasite

The parasite has a complex life cycle. Depending on the stage of development in its mammalian host, the parasite can be extracellular or intracellular. The parasite exists in different forms and shapes, possibly expressing different sets of its ~ 5000 gene pool (37) at a particular time. The parasite also has different localizations during development, infecting different cell types. Hence, various innate and adaptive immune mechanisms are involved in parasite control and elimination (38, 39). Thus, for any vaccine development, it is important to know the protective immune mechanisms to induce.

### Immunity to the Pre-Erythrocytic Stage

During the pre-erythrocytic stage, antibodies can (i) inhibit sporozoite motility in the dermis or in the liver (40), (ii) bind to sporozoite and facilitate phagocytosis by monocytes or macrophages in the spleen or the liver (41), (iii) block sporozoite invasion into hepatocytes by preventing the sporozoite ligand to interact with the hepatocyte receptor(s) (42), and (iv) inhibit sporozoite development inside the hepatocytes (42). Antibodies can also recognize parasite neo-antigens such as heat-shock protein expressed at the surface of infected hepatocytes and induce liver parasite killing through an antibody-dependent cell-mediated mechanism likely involving Kuppfer cells or NK cells (43). The production of high levels of antibodies is dependent of CD4 T cell help, preferably by recognizing (an) epitope(s) present in the sporozoite to facilitate boosting of the antibody during natural infection with the parasite. When the parasite is inside the hepatocytes, it can become the target of CD4 or CD8 T cells (44–46). Hepatocytes express MHC Class I and Class II molecules that can be loaded with parasite antigen-derived epitopes following the TAP or the endosomal pathways (44–47). T cells kill the parasite either directly or through the action of cytokines, such as IFN-γ. IFN-γ induces the inducible nitric oxide enzyme to produce nitric oxide which directly kills the liver parasites (48–51). Innate immune mechanisms involving type I interferon pathway induced by the parasite infection and active against late schizonts or against reinfection have been recently uncovered (52–54).

### Immunity against the Erythrocytic Stage

Adaptive immunity against the blood stage is more complex than in the liver stage. Merozoite-specific antibodies can (i) prevent merozoites from invading RBC alone (55–57) or, in conjunction with complement factors, (ii) prevent merozoite egress from RBC, (iii) agglutinate released merozoites, (iv) promote phagocytosis of merozoites, and (v) facilitate clearance of infected RBC (iRBC) by phagocytic cells through a mechanism called antibody cell-dependent inhibition (ADCI) (58). In ADCI, anti-merozoite cryophilic (IgG1 or IgG3) antibodies bind to merozoites and the immune complexes stimulate phagocytes to release cytokines such as TNF-α, which in turn stimulate the phagocytes to produce mediators that lead to the killing of intra-erythrocytic parasites (59, 60). *Plasmodium* parasites express antigens at the surface of iRBC (61). These antigens are mainly encoded by multigene families such as the *var* (62), *stevor* (63), and *rifins* gene families (64, 65) for *P. falciparum* or the *pir* gene family for *P. vivax, P. knowlesi*, and the rodent malaria species (66). The antigens they encoded have been implicated in the cytoadherence phenotype to endothelial cells in deep tissues in order to avoid splenic clearance (67, 68). They are also involved in other adhesive phenomena, such as rosetting (the binding of an iRBC to non-infected RBC) (69–72) and agglutination (the binding to iRBC to iRBC through bridging by platelets) (73, 74). The cytoadherence abilities of the malaria parasites have been proposed to be responsible for some of the pathologies during malaria infection. Antibodies targeting the surface antigens are thought to act through preventing cytoadherence, promoting iRBC phagocytosis, or iRBC agglutination (74, 75).

Antibodies targeting the parasite toxins could also protect from disease. During infection, multiple parasite toxins are released at the time of iRBC rupture. These toxins include the malaria pigment, a by-product of heme degradation by the parasite (76–78), glycophosphatidylinositol (GPI) moieties that are present in many merozoite proteins, a TatD-like DNase (79), a tyrosine-tRNA synthase (80), or lipids extracted from *P. vivax* schizonts (81). Protection from disease by anti-toxins antibodies has been demonstrated experimentally using synthetic glycans mimicking GPI (82).

T cells are also critical effectors in the immunity against blood-stage malaria infections, despite the lack of MHC antigens on the surface of iRBC. First and foremost, blood-stage parasite-specific antibodies secreted by B cells depend on CD4^+^ T helper cells enhancement for optimal production (39). Cytokines released by CD4 T cells are important for multiplication and maturation of B cells. The cytokines produced by malaria-specific T cells influence the isotype of the antibodies produced (83, 84) and thus possibly affecting the type of antibody-mediated responses induced. It has been shown that ADCI against *P. falciparum* is mediated by human IgG3 (induced by a Th1 response) and antagonized by IgG2 and IgG4 (induced by a Th2 response) (59, 85). Inducing the right isotype is thus important for an antibody-based vaccine. Recent studies have shown that inducing an immune response skewed toward the IgG3 could be achieved through the use of the right adjuvant (86). CD8 T cells were once thought to have only a minimal role in blood-stage immunity (87, 88). However, there is now evidence that these cells can inhibit blood-stage infection (89, 90). In particular, IFNγ-secreting CD8 T cells are important for preventing chronic blood-stage infection in mice (91).

### Immunity against the Sexual Stages

The sexual forms of *Plasmodium* parasites, gametocytes, are also targets of the immunity against the disease. They are targeted by antibodies which can induce complement-mediated killing of the gametocytes in the host blood (92, 93). In the mosquito, antibodies can (i) prevent gamete fusion (94), (ii) induce complement-killing of gametes or ookinetes (95), and (ii) prevent ookinete motility, penetration of the midgut wall and formation of oocyst (96–99). Sexual-stage parasite-specific antibodies depend on CD4 T helper cells for optimal production. However, although antibodies specific for gametes or ookinetes can be produced by vaccination, the humoral immune response cannot be boosted by repeated infections since these forms are not present during infection in the mammalian hosts. Monocytes/macrophages stimulated during infection by the parasites produce cytokines such as TNF-α which in turn stimulates the monocytes/macrophages to produce nitric oxide, a potent inhibitor of gametocytes (100).

## Mechanisms of Immune Evasion

To avoid being eliminated by the host immunity, the parasite has developed many escape strategies (Table 1).

**Table 1:** **Host immunity and parasite immune escape strategies**.

Host immunity	Parasite stage	Parasite evasion strategy	Outcome
Complement	AS, S	Bind complement regulatory factor, factor H Disrupt c-Jun kinase pathway	Prevent complement activation system Avoid recognition by complement
Monocytes/macrophages	AS	Subvert or kill phagocytes by (1) inhibiting phagocytosis and (2) inducing apoptosis	Prevent parasite elimination by (1) inhibiting phagocyte function and (2) reducing phagocyte numbers
Dendritic cells (DC)	AS	Subvert or kill DC by (1) inhibiting DC maturation and (2) engaging apoptosis receptor, Fas	Decrease DC functions by (1) preventing T cell priming and expansion and (2) inducing immunosuppression through decreased pro-inflammatory (IL12) cytokine production and increased immunosuppressive (IL10) cytokine production
Antibodies/B cells	Spz, AS, S Spz, AS, S AS AS AS AS AS Spz, AS, S AS AS	Antibody enhancement invasion and/or growth Antigen polymorphism Antigenic variation Antigenic diversion Epitope masking Smoke-screen strategy B cell dysregulation Homology with host proteins B cell apoptosis Redundancy in cell invasion pathways	Expansion of parasite in host Avoid recognition by antibody Avoid recognition by antibody Prevent the action of neutralizing antibodies Prevent the action of neutralizing antibodies Avoid antibody recognition by diverting neutralizing antibody from their target Poor or limited B cell memory Poor or no antibody response by inducing immunological tolerance Poor antibody response Allow continued invasion and expansion of parasite even when one invasion pathway is inhibited by antibody
T cells	LS AS AS AS	T cell epitope polymorphism Apoptosis Induction of expression of check-point inhibitors Regulatory T cells	Avoid T cell recognition prevent T cell priming and activity, and interfere with memory T cell development Poor T cell response Anergy and/or T cell exhaustion Negative regulation of immune responses
Hepatocytes	LS	Cellular shelter	Avoid immune surveillance due to intracellular niche

*Spz, sporozoite; LS, liver stage; AS, asexual blood stage; S, sexual stage*.

## Evasion of the Complement System

The complement system is one of the first innate immune defense mechanisms against pathogens. Many proteins are involved in the activation or the regulation of its lytic activity (101). During the blood-stage, the parasite has developed multiple ways of evading the action of complement. *P. falciparum* merozoites and iRBC bind to the factor H (fH), a complement regulator factor, and its alternatively spliced form fH-like protein 1 through its surface molecule Pf92. In the mosquito, gametes bind the fH through PfGAP50 (102). In both situations, this allows protection against the activation of complement-mediated lysis. In addition, ookinetes express Pfs47, which disrupts the c-Jun N-terminal kinase pathway and prevents mosquito midgut epithelial nitration, making the parasite undetectable by complement system (103).

## Antibody-Dependent Enhancement of Infection

Antibody-dependent enhancement of infection was first described for viruses (104). Similarly, antibody-dependent enhancement of infection has been described for all stages of parasite development in the mammalian host. It was first reported in the early 1990s that antibodies against the repeat region of the CS protein enhance sporozoite entry and development in hepatocytes for both rodent (42, 43) and human parasites (105). Antibodies against the *P. falciparum* asparagine-rich protein enhance merozoite invasion of RBC *in vitro* (106). Merozoite-specific antibodies in conjunction with complement can also facilitate RBC invasion (107). Antibody-dependent enhancement of infection was also observed *in vivo* after immunization with a *Plasmodium berghei* parasite blood-stage preparation which led to increased death after challenge (108). In the sexual stage, anti-gamete antibodies were shown to enhance transmission to the mosquito (109).

Antibodies against proteins expressed in one stage of the parasite’s development might mediate an enhancement effect in another stage of the parasite’s development. This was observed for antibodies against two antigens, the CS protein and heat-shock protein 1 (HSP-1) (110). The CS protein is expressed during sporogony in the mosquito and in the subsequent sporozoite stage. Do Rosario et al. showed that sporozoites generated during sporogony in the presence of anti-CS protein antibodies cannot be inhibited by human polyclonal anti-CS (sporozoite CS) protein (111). HSP70-1 is expressed by the liver stage and the blood stages (43, 110). HSP70-1 antibodies have no effect against the blood-stage parasites but they can partially mediate inhibition of liver-stage development through ADCI (43). However, vaccination with a recombinant protein against the C-terminal fragment of HSP70-1 increased the number of gametocytes generated and the subsequent transmission to mosquito (112). Thus, it was proposed that antibodies against the liver-stage parasites can drive merozoites to differentiate into gametocytes and facilitate transmission (112). Immune cross-talk between the different stages of the infection has not been extensively studied beyond the two reports mentioned above. This cross-talk has wider implications. Indeed vaccination against one stage may influence the subsequent stages positively or negatively. For any vaccine against the pre-erythrocytic or blood stages, there will be a need for follow-up studies on the effect on transmission and development of the parasite in the mosquitoes.

## Antigenic Polymorphism

Similar to all other organisms, the parasite is prone to mutation since the replicative machinery is not error free. In its mammalian host, the parasite is haploid and the mutation rate is ~1–0.7 × 10^−9^ mutations per base per generation (113). Since the *Plasmodium* parasites have a 24- to 72-h blood cycle, there is a high probability that mutation can occur and generate different parasite clones. In the mosquito host, the parasite undergoes sexual reproduction where two haploid gametocytes will generate four haploid sporozoite progenies. Recombination does occur in the mosquito stage, and, thus, it will increase the occurrence of gene polymorphism. For some antigens, hundreds of haplotypes have been observed (114). Polymorphism in the coding sequence can be due to point mutations, insertions, or deletions. Interestingly, many *Plasmodium* antigens possess regions of repeats which can vary in size and number of repetitive units (115). Such diversity is, in most cases, the result of immune pressure since mutations often occur in regions which can be recognized by antibodies or T cells. Mutations in B epitopes abolish the recognition of parasites by antibodies and may lead to the selection of parasites with a different haplotype. This is important for vaccine development since antibody-based vaccine formulation targeting polymorphic epitopes may either have limited efficacy or select for vaccine-resistant parasite. One such example is the vaccine based on the AMA-1 protein, a highly polymorphic antigen (116–118). However, it is possible to envisage that immunogen(s) inducing broadly inhibitory antibodies recognizing multiple variants may circumvent polymorphism. Studies to identify structural conserved constraints in different variants may pave the way for new vaccines against polymorphic antigens (56).

Polymorphism in T cell epitopes may have profound consequences in T cell responses and have shown to limit the efficacy of the RTS,S vaccine (119). T cell epitopes are, in general, 8–11 amino acid (aa) and 11–25 aa in length for CD8 T cell and CD4 T cells, respectively (120). After the digestion of parasite protein in the cytoplasm, CD8 antigenic peptides are generated and transported to endoplasmic reticulum by the transporter antigenic peptide protein in the endoplasmic reticulum where they are loaded onto MHC class I molecules and beta 2-microglobulin. CD4 epitopes are generated in endosomes after phagocytosis and protease digestion where they meet MHC class II. Peptide–MHC complexes are then expressed in the surface. Exogenous antigens can also be presented by cross-presentation by MHC class I (121). These complexes can be recognized by the T cell receptor (122). Mutations in the amino acids that anchor the epitope to the MHC grove can prevent binding of the peptides to the MHC or recognition by the TCR and thus abrogate T cell activity (123, 124). This was demonstrated for the CS protein (125). Altered peptide ligand (APL) can still bind to the MHC molecules; however, they prevent T cell proliferation, no matter if it was used singly or concurrently with a wild-type peptide. They can also prevent cytokine production (126) or change their production pattern, i.e., from IFN-γ to the immunosuppressive cytokine interleukin-10 (127). These APL can also interfere with induction of memory T cells from naïve T cells (128). This potent mechanism of immune escape is a major hurdle for vaccine development. For any T cell-based vaccine to succeed, a thorough analysis of T cell polymorphism and their effect should be performed.

## Antigenic Variation

Antigenic variation was first described by Neil Brown during *P. knowlesi* chronic relapsing infection in Rhesus monkeys induced by sub-curative drug treatment (129). In an agglutination assay, antibodies produced against different relapse parasite broods agglutinated only schizonts of the immunizing brood but not the other broods. It was further shown that antibodies (130) and spleen could induce antigenic variation in Rhesus monkey infected with a cloned line of *P. knowlesi* (67) or monkey infected with cloned lines of *P. falciparum* (131). The latter experiments suggested that, contrary to antigenic polymorphism where parasites have different alleles and can be categorized as genomic clones, antigenic variation is a phenotypic variation occurring with the same genomic clone of the parasite. The molecular basis of this phenomenon was elucidated with the sequencing of large segments of the *P. falciparum* and *P. knowlesi* genomes and the subsequent complete sequencing of the genome of many *Plasmodium* species. Antigens prone to antigenic variation are often expressed on the surface of iRBC. Examples includes the multigene family, such the *var* genes (62), the *stevor* gene family, the *rifin* gene family (63), the *surfin* gene family (132), the *sicavar* gene family (133), and the *Plasmodium* interspersed repeats (*pir*) genes. The *var* gene family is the most studied and has been shown to be the target of protective anti-blood-stage antibodies (134, 135). It is also involved in many of malaria pathologies due to parasite sequestration resulting from iRBC cytoadherence (136). PfEMP1 proteins, encoded by the *var* gene family, are highly polymorphic and have different variable domains, called Duffy binding-like domain, which determine their binding specificities to various ligands on endothelial cells such as thrombospondin (137), CD36 (138), ICAM-1/CD54 (139), chondroitin sulfate A (140–142), VCAM-1, E-selectin (143), αvβ3 integrin (144), hyaluronic acid (145), PECAM-1/CD31 (146), gC1qR/HABP1/p32 (147), or endothelial protein C receptor (148). PfEMP-1 proteins bind also to RBC through complement receptor 1 to form rosettes (149). *P. falciparum* parasites possess ~60 var genes distributed over the 14 chromosomes of the parasite (37). *Var* genes expression is extremely regulated and only one PfEMP1 type is produced and displayed on the surface of iRBC (150). At each cycle, the parasite switches the expression of the *var* gene at a rate of 2% *in vitro*, generating new clones with new antigenic and adhesive phenotype (151, 152). Vir proteins, members of a superfamily of the *pir* multigene superfamily, also mediate cytoadherence of *P. vivax* iRBC to endothelial cells (153), resulting to sequestration of mature iRBC (154). There are ~350 *vir* genes, and they are also highly polymorphic (155, 156). Stevor and Rifin proteins are involved in cytoadherence processes and are also highly polymorphic (70, 71). In the recent years, it has become clear that some members of these multigene family has a particular role and may be involved in certain pathology. As example, *var2*CSA mediated the specific cytoadherence of iRBC to placenta (142) and is associated with placental pathology, such as still birth and fetus growth retardation Vaccination using immunogens based on *var2*CSA will thus induce inhibitory antibodies, preventing cytoadherence (157, 158) and placental sequestration. Thus defining the role of members of these multigene families may lead to tailored immune intervention.

## Antigenic Diversion

Antigenic diversion occurs when non-inhibitory anti-parasite antibodies prevent the action of inhibitory antibodies. Antigenic diversion has been observed with the merozoite surface protein (MSP)-1. MSP-1 is a surface protein which binds to glycophorin A, a molecule present on the surface of RBC, and thus is essential for merozoite invasion (159, 160). MSP-1 is cleaved at the time of invasion. Neutralizing antibodies, which block the proteolytic cleavage in the C-terminal part (MSP-1_19_) of the protein (antibodies), can prevent invasion (152, 161). However, antibodies against the adjacent or overlapping region can block the effect of MSP1_19_-inhibitory antibodies (blocking antibodies) and thus allowing invasion to occur (162). One possibility to overcome this phenomenon is to design immunogen(s) that either induce(s) neutralizing but not blocking antibodies in naïve individuals or tip the balance to greater production of neutralizing antibodies over blocking antibodies in naturally exposed individuals. So far, there has been limited success with MSP-1 (159, 163).

## Epitope Masking

Epitope masking is the capacity of non-parasite-specific antibodies to prevent parasite-specific inhibitory antibodies to react with their epitopes. During the establishment of an antibody response, IgM precedes IgG production and thus are the first line of humoral response either alone or in combination with complement (164). Malaria-specific IgM have been shown to have efficient inhibitory activity against sporozoite and iRBC (165). However, IgM with different specificities can also bind to PfEMP-1 molecules expressed at the surface of iRBC through their Fc portion (Fc) (166). Non-parasite-specific IgM (NpsIgM) promote rosetting and thus may facilitate sequestration, in order to avoid splenic elimination. NpsIgM binding to the PfEMP-1 VARCSA2, which is involved in the binding of iRBC to chondroitin sulfate in the placenta, appears to protect iRBC from phagocytosis mediated by IgG (167). NpsIgM also binds to the MSP DBLMSP and DBLMSP2 and prevents IgG binding to these molecules by masking epitopes (168). The role of these two molecules in merozoite biology is still unclear but non-specific IgM epitope masking may be important for protecting the parasite against a specific IgG inhibitory response.

## Smoke-Screen Strategy by Cross-Reactivity

Smoke-screen strategy is often used by the parasite to divert the antibodies specific for one antigen (antigen A, e.g., an epitope of inhibitory antibodies) to react against another antigen (antigen B, e.g., an epitope of non-inhibitory antibodies) which possess regions homologous to antigen A. Antibody reactivity to segments shared by the two proteins will decrease the amount of antibodies reacting to antigen A and thus reducing efficacy of inhibitory antibodies that can inhibit parasite invasion or development. Many *Plasmodium* antigens contain repeats, and it was shown that cross-reactivity can occur between different blocks of repeats (169). Cross-reactivity has been observed: (i) between different epitopes from the same block of repeats [i.e., within the same block of repeats in the CS protein, or Ring-erythrocyte surface (RESA), or S-antigen or falciparum interspersed repeat antigen (FIRA)]; (ii) between epitopes present in different blocks of the same antigen (i.e., RESA or FIRA), between repeats of different antigens [between the CS protein and the cross-reactive antigen (CRA), between the histidine-rich proteins]. So far, the importance of this mechanism in the immune response evasion has remained uncertain.

## Dysregulation of B Cells

Atypical memory B cells are a population of hypo-responsive memory cells which have been first described in chronic HIV infections (170). They increase in numbers during chronic exposure to malaria and are poor responders to antigen restimulation (171). However, they are able to produce neutralizing antibodies (172). These cells might be important during repeated stimulation due to constant exposure and help to control parasite density. However, in regions with lower exposure or in the absence of reinfection, immunity might wane rapidly.

As mentioned in the previous section, many *Plasmodium* antigens contain repeats. Similar to many viral antigens (173) and haptenated polymers (174), repeats-containing *Plasmodium* antigens can stimulate B cell independently of T cell help. However, this often leads to the production of mainly IgM and limited B cell memory (175). In addition, exposure to antigens containing repeated motifs can result in the suppression of an ongoing T cell response (176, 177).

## Homologies with Host Proteins

Many *Plasmodium* antigens involved in the invasion of host cells have regions which strong homologies with host proteins involved in protein–protein interactions. Thrombospondin type-1 repeat (TSR) domains and von Willebrand factor (vWF)-like A domains are present in the CS protein, thrombospondin-related anonymous protein (TRAP) (178, 179), CS protein TRAP-related protein (CTRP) sporozoite surface protein (180), secreted protein with an altered thrombospondin (SPATR) (181), and thrombospondin-related apical merozoite protein (TRAMP) (182). These molecules are involved in different stages of parasite development, in sporozoite and merozoite motility, and in invasion of mosquito midguts, salivary glands, hepatocytes, and RBC. Many merozoite proteins such as MSP-1, MSP-4, MSP-5, MSP-8, and MSP-10 (183), PfRipr (184) and sexual-stage protein, such as P25 or P28 (185, 186), have been found to contain an epidermal growth factor domain that is involved in its binding to their receptor. Due to the homology to the host protein, the induction of antibodies to epitopes contained in these homologous regions is difficult since the host is tolerized against its own proteins. Immunization with immunogens containing these motifs with strong adjuvants could possibly escape immunological tolerance but may have the risk of inducing auto-immunity.

## Immunosuppression

Myeloid cells are essential mediators of an efficient immune response against the malaria parasites. Monocytes, macrophages, and neutrophils phagocytose iRBC, which eventually leads to the elimination of the parasites (187). Phagocytosis by monocytes/macrophages can be mediated through the interactions of PfEMP-1 and CD36 without inducing or increasing a protective pro-inflammatory response (188). However, during infection, phagocyte functions can be diminished after the ingestion of the malaria pigment or hemozoin (the digested product of hemoglobin by the parasites) (189, 190). Pigment-loaded macrophages cannot phagocytose more iRBC, and their capacity to generate radical oxygen intermediates is also reduced (191).

Dendritic cells (DCs) are essential to induce adaptive immune responses (192). Engagement of CD36 and CD51 (the αv integrin chain) on DCs by PfEMP-1-expressing parasites impair DC maturation and its capacity to prime T cell responses, leading to reduced production of pro-inflammatory cytokines such as IL-12 and increased production of immunosuppressive cytokines such as IL-10 (193).

Acute blood-stage infection induces a strong activation of mononuclear cells. This can result in apoptosis in monocytes, B cells, T cells, and DCs (194–199). Acute infection can also lead to thymus apoptosis and depletion, thereby diminishing the output of new naïve T cells (200). On the other hand, chronically activated T cells (both αβ and γδ) during lingering blood infection or multiple reinfections can also enter a phase of anergy (201–203) and/or exhaustion (204). *Plasmodium* infection activates check-point inhibitors molecules such as program cell death protein 1 (PD-1), program death ligand 1 (PD-L1), PD-L2, lymphocyte-activation gene 3 (LAG-3), and cytotoxic T lymphocyte antigen-4 (CTLA-4) in CD4 and CD8 T cells. In rodent models, blockade of these molecules facilitates the elimination of blood parasites (91, 205, 206) and the establishment of T cell memory (207). Regulatory T cells often expand during infection (208). Their principal role is to control the excessive pro-inflammatory response which depending on the parasite species may be beneficial or detrimental (209–211). Taken together, these mechanisms leading to immunosuppression are likely to favor survival of the parasite in the host and prevent the establishment of an efficient memory response. In addition, while it might also be beneficial for the host to prevent immunopathogenesis (212), ensuring host survival is advantageous to parasite survival.

## Redundant Pathways of Red Blood Cell Invasion

Invasion of RBC by merozoites is a complex process. It involves a precise and coordinated expression of different sets of proteins which are either expressed on the surface of the merozoite or in the organelles such as rhoptries, micronemes, and dense granules located at the apical end of the merozoite (183). The invasion process has been amply studied for *P. falciparum*. It was shown that the parasites can use multiple pathways involving at least nine ligand/receptor combinations to invade a RBC. There are two parasite protein families involved in the host-cell selection and invasion process; the reticulocyte-binding protein homologs (Rh) and the erythrocyte binding-like proteins (EBL) (213). These proteins are polymorphic, and their expression varies depending on parasite clones. Polymorphisms in a *P. falciparum* EBL have been shown to result in a change in their receptor specificity (214) or in a switch to Rh-dependent invasion, a different invasion pathway for RBC invasion (215). Variation in the use of erythrocyte invasion pathways results in evasion of human inhibitory antibodies (216). Thus, this suggests that vaccines targeting only one pathway will select parasite using other pathways. However, so far, the pathway involving Rh5 and its ligand Basigin/CD147 has been shown to be required for all parasites clones and isolates tested so far and has shown limited polymorphism (217). It thus represents a promising vaccine candidate (218, 219).

Contrary to *P. falciparum, P. vivax* was thought to use only one pathway to invade RBC (220). *P. vivax* merozoites only invade reticulocytes, the immature RBC. Since people in West Africa or with West African origins naturally lack the Duffy antigen, they are resistant to *P. vivax* blood infection (221, 222). This serves as a strong argument for the existence of only one invasion pathway for *P. vivax*. However, in the recent years, many studies have reported *P. vivax* infections in Duffy-negative patients (220, 222–228), suggesting that *P. vivax* merozoites may use another pathway to invade reticulocytes (229). This may limit the efficacy of vaccine based on the Duffy-binding protein, the ligand of the Duffy antigen (230), by selecting for parasites that are able to invade *via* this alternative pathway.

## Shelters

Infection with sporozoites from some human/primate parasites, such as *P. vivax, P. ovale, P. cynomolgi, Plasmodium simiovale*, and *Plasmodium fieldi*, may lead to relapse (231). After a patent blood infection is completely cleared by the immune system or by drug treatment, blood parasites may reappear and induce new clinical attacks (232). This new infection originates from long-lasting liver forms, called hypnozoites (233). These non-dividing and metabolically active forms (234) can persist in the liver of their infected hosts for long period of time. Histological analysis on liver from infected rhesus monkeys did not show any signs of cellular immune responses against hypnozoites or late-developing schizonts originating from these forms (235, 236). This suggests that hypnozoites do not trigger any immune response, and do not lead to the expression of MHC molecules containing parasites-derived peptides. Due to the difficulty in studying these forms *in vitro* and *in vivo* (237), the mechanisms by which the parasite subverts immune recognition is unknown. Vaccine development against the pre-erythrocytic of *P. vivax* has been limited, and formulations targeting the liver forms may not work against the hypnozoites.

As mentioned above, during primary sporozoite-induced infection in natural hosts, no cellular immune responses are observed (8, 238–240). The liver is rich in macrophages and Kupffer cells, which can phagocytose emerging hepatic merozoites. To avoid being recognized by phagocytes, merozoites are released inside vesicles called merosomes in to the sinusoid lumen. Merosomes do not express macrophage-recognition signal such as phosphatidyl-serine and thus escape being phagocytosed (8). Merosomes are carried to the lung vasculature where blood circulation is low (241) before releasing their merozoite cargo (242).

Vesicles containing merozoites have been observed inside other cell types other than RBC, such as platelets (243), in macrophages (244), and in DCs (245, 246). It has been shown that merozoites can also divide in the DCs expressing CD317/tetherin, and eventually initiate new infections (245). It is not yet known how the phagocytosed parasites evade digestion or if a population of merozoites can infect and multiply in these cells. Free merozoite-containing vesicles, called merophores, have also been observed in the lymph circulation during rodent malaria infection (247, 248). This could explain recrudescence or latency of infection. However, as for the merosomes, these vesicles may be devoid of recognition signals for phagocyte uptake and may not express parasite antigens on their surface.

## Conclusion

Malaria parasites have been interacting with its mammalian hosts for more than 150 million years (249) and have efficiently evolved to survive under the pressure of the host immune system. The parasite has developed numerous immune evasion mechanisms. Since new host immune mechanisms against the parasite are constantly being discovered, it will be of no surprise that new immune escape mechanisms by the parasite will be uncovered. For example, there is still little known on how innate immunity are induced during infection and on how protective epitopes are generated. The ultimate goal of understanding the immune responses to the malaria parasites is the development of vaccines. The selection of antigens and delivery system is governed by the target. Historically, before the whole genome was sequenced, the first malarial antigens, that were cloned and sequenced, have been assumed to be good vaccine candidates (250). However, all these antigens are immunodominant and are involved in immune escape. For vaccines to develop in a timely manner, the selection criteria should involve a more stringent GO-NO Go selection based on the analysis of the potential of the vaccine candidate to avoid immune escape. This also calls for an immunological approach to define correlates of protection to guide vaccine development. The development of the experimental human malarial challenge model, where complete sterile protection can be obtained (34, 35), is strong evidence that a vaccine against malaria can be obtained. Together with parasite genetics, the development of the rodent models and the experimental human challenge model would greatly assist in making the critical GO-NO Go decisions and facilitate the development of an efficacious vaccine against malaria in the foreseeable future.

## Author Contributions

Both the authors listed have made substantial, direct, and intellectual contribution to the work and approved it for publication.

## Conflict of Interest Statement

The authors declare that the research was conducted in the absence of any commercial or financial relationships that could be construed as a potential conflict of interest.
